# The use of the concept of transition in different disciplines within health and social welfare: An integrative literature review

**DOI:** 10.1002/nop2.249

**Published:** 2019-03-06

**Authors:** Ulrika Lindmark, Pia H. Bülow, Jan Mårtensson, Helén Rönning, A.D.U.L.T. Research Group, Inger Ahlstrand, Anders Broström, Eleonor I. Fransson, Bengt Fridlund, Nina Gunnarsson, Maria Henricsson, Sofia Kjellström, Anna Sandgren

**Affiliations:** ^1^ Department of Natural Science and Bio Medicine, Center for Oral Health, School of Health and Welfare Jönköping University Jönköping Sweden; ^2^ Department of Social Work, School of Health and Welfare Jönköping University Jönköping Sweden; ^3^ Department of Social Work University of the Free State Bloemfontein South Africa; ^4^ Department of Nursing, School of Health and Welfare Jönköping University Jönköping Sweden; ^5^ School of Health and Welfare Jönköping University Jönköping Sweden

**Keywords:** literature review, nursing theory, occupational therapy, oral health, social welfare, social work, theory–practice gap, transition

## Abstract

**Aims:**

To continuing the quest of the concept of transition in nursing research and to explore how the concept of transition is used in occupational therapy, oral health and social work as well as in interdisciplinary studies in health and welfare, between 2003–2013.

**Design:**

An integrative literature review.

**Methods:**

PubMed, CINAHL, PsycINFO, DOSS, SocIndex, Social Science Citation Index and AMED databases from 2003–2013 were used. Identification of 350 articles including the concept of transition in relation to disciplines included. Assessment of articles are in accordance to Meleis' typologies of transition by experts in each discipline. Chosen key factors were entered into Statistical Package for the Social Sciences (SPSS).

**Results:**

Meleis' four typologies were found in all studied disciplines, except development in oral health. The health‐illness type was the most commonly explored, whereas in social work and in occupation therapy, situational transitions dominated.

## 1 INTRODUCTION

In nursing research, *transition* has been described as the “passage from one life phase, condition, or status to another,” as “periods in between fairly stable states” (Chick & Meleis, 1986, p. 238) and as “processes that occur over time,” which can be divided “into stages and phases” (Schumacher & Meleis, 1994, p. 121). From the work of various researchers, transition has been presented as a central concept in the discipline of nursing for the last four decades, (Meleis, 1975; Suva et al., 2015). During this period of time, the components of transition have been identified and described (Chick & Meleis, 1986), expanded by one additional typology (Schumacher & Meleis, 1994) and the concept has been further extended and redefined (Meleis, Sawyer, Im, Messias, & Schumacher, 2000)—all in nursing research.

The prominent figure in defining transition in nursing research is Afaf Ibrahim Meleis, who first wrote about the concept in relation to role insufficiency and role supplementation (Meleis, 1975). In addition to being educated in nursing, Meleis has a graduate education in sociology, as well as medical and social psychology (Im, 2010), which probably influenced the development of the transition theory. Meleis drew on theories that are now considered as classical sociological theoretical approaches, such as role theory (Turner, 1962) and symbolic interactionism (Blumer, 1969). Additionally, Kralik, Visentin, and Van Loon (2006) noted anthropology as the discipline where transition historically has been described. This makes the concept of transition adaptable and interesting also to other disciplines in health and welfare such as occupational therapy, oral health and social work (Munck, Björklund, Jansson, Lundberg, & Wagman, 2018).

Transition, in the mentioned research areas, is not highlighted as the main element of a theoretical framework, as in nursing. However, in sociology and in relation to illness, the phenomenon of transition has been captured by concepts such as *career* (Davis, 1963; Roth, 1963), *change* (Watzlawick, Weakland, & Fisch, 1974), *illness trajectories* (Glaser & Strauss, 1968) and *biographical disruption* (Bury, 1982). Nevertheless, transition as a concept has been used in health and social welfare since at least the 1930s (Proehl, 1938).

### 1.1 Background

To demonstrate the diversity of the concept in nursing, four typologies of transition are described: developmental, situational, health‐illness and organizational (Chick & Meleis, 1986; Schumacher & Meleis, 1994). The *developmental* category involves responses of stages in the life cycle such as parenthood, mainly focusing on the individual perspective. Meleis (1975) emphasized two kinds of developmental transitions, which in particular are associated with health problems—the transition from childhood to adolescence and the transition from adulthood to old age. *Situational transitions* consist of various changes in educational and professional roles (Schumacher & Meleis, 1994). Other studied situational transitions are changes in the family, for example widowhood, or transitions due to migration, homelessness and leaving an abusive relationship. *Health‐illness transitions* focus on how individuals and families experience different illness contexts but also on transitions among levels of care during the course of illness (Schumacher & Meleis, 1994). In their work, Schumacher and Meleis, (1994 added *organizational transitions* as the fourth type of transition, which represents “changes in the wider social, political, or economic environment or by intraorganizational changes in structure or dynamics” (p. 21).

In an attempt to broaden the knowledge of the concept, Kralik et al. (2006) reviewed articles from diverse professional fields published between 1994–2004, where transition was used. They described these as “health literature” but also included a social focus. In their review, only research with qualitative methodologies was included.

The concept of transition, as described in nursing, is complex and multidimensionally identified as awareness, engagement, change and difference, time span, critical points and events (Meleis et al., 2000). Future research might identify other factors important for understanding the complexity of transition to prevent and minimize risks for unhealthy transitions. This research will probably be found in other disciplines in health and welfare, which provides an opportunity to discover transition processes in other diverse populations and contexts as suggested (Meleis et al., 2000). The concept of transition embodies an important issue and seems currently to be commonly used in various fields outside nursing. However, to the best of our knowledge, the concept is not explored and reviewed in relation to other disciplines in the field of health and social welfare.

## 2 AIM

To continuing the quest of the concept of transition in nursing research and to explore how the concept of transition is used in occupational therapy, oral health and social work, as well as in interdisciplinary studies in health and welfare, between 2003–2013.

Research questions:

- Are the four typologies (developmental, situational, health‐illness and organizational) elaborated by Meleis et al. (2000) applicable for the studied disciplines and if, which differences and/or similarities are found?
- Where contexts are the concept of transition used in the various disciplines?
- Who underwent the transition and from what perspectives are the transition regarded in different disciplines?

## 3 METHOD

### 3.1 Design

This integrative literature review (Whittemore & Knafl, 2005) of transition as a concept was used to examine the literature in various disciplines in health and welfare.

### 3.2 Inclusion and exclusion

The primary criteria for inclusion in this review were scientific articles published between 2003–2013, original papers written in English and articles that illuminated the concept of transition as a part of the results in the context of nursing, oral health, occupational therapy, social work, or interdisciplinary studies in health and social welfare. Exclusion criteria were if the participants were predominantly <18 years old, if it was a review article, if there was a meeting abstract or if the abstract was missing, if the article did not focus on transition and if the transition concerned another area than the above‐mentioned disciplines or did not focus on the outcome of the person(s) in transition.

### 3.3 Literature search

This literature review used PubMed, CINAHL, PsycINFO, DOSS, SocIndex, Social Science Citation Index and AMED databases. The following Medical Subject Heading (MeSH) terms were used in the search: Transition AND nurs*, OR transition AND car*, OR transition AND social work*, OR transition AND social car*, OR transition AND occupational therap*, OR transition AND oral health*, OR transition AND dental hygien*. To make the data manageable, each search was performed for both title and abstract. If the number of articles in one search for abstracts (e.g., transition AND nurs*) exceeded 300, the number of hits in the title was selected instead.

### 3.4 Search outcome

After duplicates (*N* = 2,365) were removed, 2,523 unique references were found in the databases and were screened (PubMed 580, CINAHL 132, PsycInfo 255, DOSS 119, SocIndex 381, Social Sciences Citation Index 882, AMED 174).

### 3.5 Article selection

All retrieved titles and abstracts were screened to determine the eligibility by an interdisciplinary review team of 11 researchers, all an “expert” in at least one of the studied disciplines. The interrater reliability was tested in two steps. First, 50 articles were assessed using a template as fulfilling the criteria, doubtful fulfilling the criteria or as not meeting the criteria. Good concurrence was reached in 68%. In step two, after a discussion of the reviewed articles and the differences in grading, 25 articles were assessed in a similar way and concurrence was reached in 84%.

**Figure 1:**
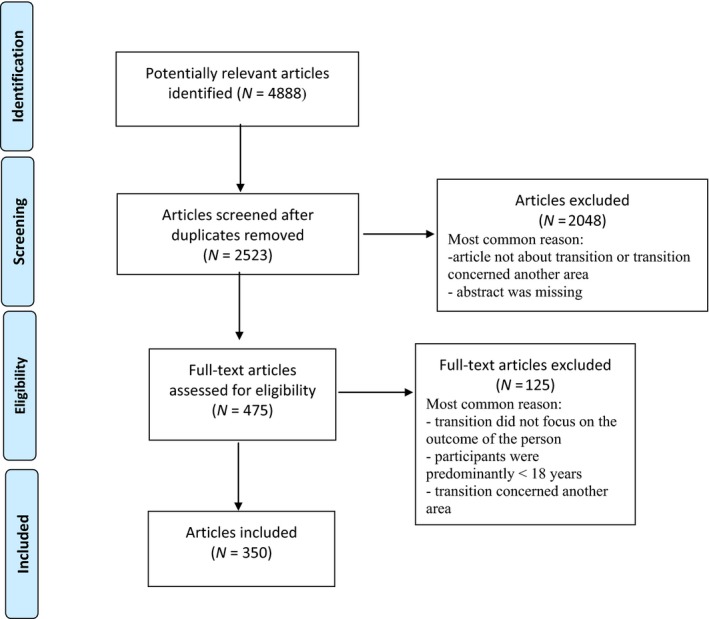
Flow diagram of the systematic review process

After this, the abstracts (*N* = 2,523) were distributed to the review team (200–250/reviewer) in accordance with each researcher's expertise to be assessed according to the inclusion and exclusion criteria. Most excluded articles were primarily based on transitions outside the health and welfare sector. After this screening, 475 full‐text articles were assessed for eligibility and 125 of them were excluded, resulting in 350 articles included for analysis (Figure 1).

### 3.6 Analysis

**Figure 2:**
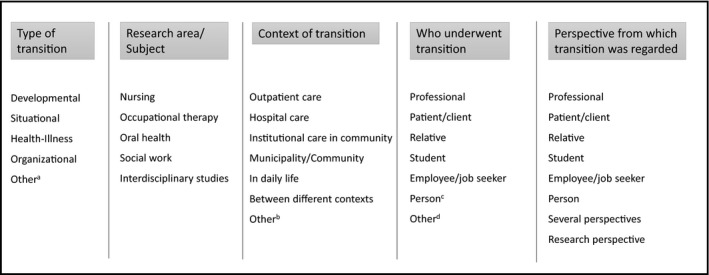
Key factors from the included articles which formed the variables included in the analysis. (a) Other: type of transition other than those described by Meleis et al. (2000) or included in more than one type. (b) Other: mixed research context or undefined context. (c) Person: in this study, “person” was considered as an individual without any categorized title. (d) Other: several people or groups underwent the transition, organization or were difficult to define

To make the analytic work more systematic and manageable, chosen key factors were entered into Statistical Package for the Social Sciences (SPSS), according to variables included in the analysis (Figure 2). The results are presented regarding the typology of transition, the context of transition in studied disciplines, who performed the transition and from what perspective the transition was regarded. Presented results are exemplified by one or two articles.

### 3.7 Ethics

Research Ethics Committee approval.

## 4 RESULTS

Of the 350 articles included, most studies were conducted in North America (60%) and Europe (22%). In nursing (*N* = 106) and occupational therapy (*N* = 39), the pattern for methods used in research was similar (Table 1). Almost half of these studies used qualitative methods (mainly interviews), about a quarter used quantitative methods (mostly questionnaires), a fifth part was described as “theoretical summaries,” and approximately 10% combined qualitative and quantitative methods. In social work (*N* = 42), the qualitative methods dominated and there were less theoretical summaries. In oral health (*N* = 11), no studies had a qualitative design and more than 80% used quantitative design (mostly questionnaires). In interdisciplinary research, (*N* = 152) quantitative and qualitative methods were used equally and fewer studies were described as theoretical summaries compared with other studied research areas. Table 1 describes different aspects of the concept of transition in relation to the five disciplines included in this study.

**Table 1:** The articles description of the research areas in relation to transition (*N* = 350)

	Nursing	Occupational therapy	Oral health	Social work	Interdisciplinary	Total
	*N* = 106	*N* = 39	*N* = 11	*N* = 42	*N* = 152	*N* = 350
Type of transition (%)						
Developmental	6.6	12.8	0.0	11.9	12.5	10.3
Situational	37.7	46.2	9.1	64.3	28.3	36.9
Health‐illness	41.5	35.9	54.5	9.5	51.3	41.7
Organizational	13.2	5.1	27.3	11.9	6.6	9.7
Other	0.9	0.0	9.1	2.4	1.3	1.4
Context for the transition (%)						
Outpatient care	8.5	2.6	63.6	9.5	10.5	10.6
Institutional care in hospital	13.2	2.6	0.0	0.0	13.2	10.0
Institutional care in community	4.7	5.1	0.0	7.1	10.5	7.4
Municipality/Community	12.2	18.0	9.1	14.2	7.9	11.1
In daily life	21.7	35.9	18.2	19.1	24.9	24.3
Between different contexts	39.6	25.6	9.1	42.9	31.6	34.0
Other	0.0	10.3	0.0	7.1	1.3	2.6
Who underwent the transition? (%)						
Professional	29.2	0.0	0.0	7.1	2.0	10.6
Patient/client	48.1	53.8	63.6	23.8	78.9	59.7
Relative	8.5	2.6	0.0	0.0	2.6	4.0
Student	3.8	12.8	0.0	4.8	2.6	4.3
Employee/job seeker	0.9	12.8	0.0	4.8	1.3	2.9
Person	5.7	17.9	27.3	47.6	12.5	15.7
Other	3.8	0.0	9.1	11.9	0.0	2.9
From which perspective? (%)						
Professional	39.6	20.5	9.1	21.4	19.1	25.4
Patient/client	20.8	25.6	54.5	4.8	38.2	28.0
Relative	9.4	7.7	0.0	0.0	12.5	9.1
Student	3.8	10.3	0.0	2.4	1.3	3.1
Employee/job seeker	0.0	7.7	0.0	0.0	0.7	1.1
Person	5.7	17.9	27.3	38.1	9.2	13.1
Several perspectives	12.3	10.3	0.0	19.0	18.4	15.1
Research perspective	8.5	0.0	9.1	14.3	0.7	4.9
Method (%)						
Quantitative	22.6	25.6	81.8	23.8	41.4	33.1
Qualitative	47.2	46.2	0.0	57.1	46.7	46.6
Qualitative and quantitative	10.4	10.3	0.0	9.5	3.9	7.1
Theoretical summary	19.8	17.9	18.2	9.5	7.9	13.1
Data collection method (%)						
Questionnaire	17.9	20.5	63.6	14.3	30.9	24.8
Interview	34.0	35.9	0.0	50.0	38.2	37.1
Observation	0.9	2.6	0.0	0.0	3.3	2.0
Focus group	7.5	0.0	0.0	0.0	4.6	4.3
Theory paper	19.8	10.3	9.1	2.4	6.6	10.5
Mixed method	17.0	20.5	9.1	21.4	15.8	17.1
Other	2.8	10.3	18.2	11.9	0.7	4.3

### 4.1 Typologies of transition

All articles considered as research on transition could be coded into one of the four types of transition defined by Meleis and her colleagues except five (1.4%), (Schumacher & Meleis, 1994) (Table 1). In all, health‐illness transition was the type of transition (41.7%) most often described, followed by situational (36.9%), developmental transition (10.3%) and organizational (9.7%). In oral health, interdisciplinary studies and nursing, the health‐illness type of transition was the most common (54.5%, 51.3% and 41.5%, respectively), whereas in social work and in occupational therapy, situational transitions dominated (64.3% and 46.2%, respectively) (Table 1).

*Health‐illness transitions* were most often due to removals in the healthcare system (Brennan, Spencer, & Roberts‐Thomson, 2012; Kirsebom, Wadensten, & Hedström, 2013), or the transition occurred in specialist care, such as for HIV (Fair, Sullivan, & Gatto, 2010) and in palliative care (Crighton, Coyne, Tate, Swigart, & Happ, 2008), or during a time period between in‐/outpatient care and home (Brauer, Hay, & Francisco, 2011; Foust, Vuckovic, & Henriquez, 2012).

*Situational transitions* mainly regarded conditional changes focusing on caregivers (Cook, Pierce, Hicks, & Steiner, 2006; Eriksson & Sandberg, 2008), out‐of‐home care such as foster care (Daining & DePanfilis, 2007), becoming a professional (Johnstone, Kanitsaki, & Currie, 2008; Yan, Gao, & Lam, 2013), professional practices (Höjer & Sjöblom, 2011) or retiring (Jonsson, 2011; Manski et al., 2011).

*Organizational transitions* illuminated, for instance, programs or strategies to facilitate the changeover from newly qualified professionals to practice (Boehm & Tse, 2013), social changes in the society (Monden, 2005) and improvements (Page, Martin, & Loeb, 2004), or changed working roles and routines (Damianakis, Climans, & Marziali, 2008).

*Developmental* transitions mostly concerned individual development such as the transition from childhood to adulthood (Wilson, Cunningham‐Burley, Bancroft, & Backett‐Milburn, 2008), transitions during menopause (Mishra, Brown, & Dobson, 2003), becoming a parent (Klingberg‐Allvin, Binh, Johansson, & Berggren, 2008; Reeves, 2006) or different transitions in late middle‐age (Salovaara, Lehmuskallio, Hedman, Valkonen, & Näsänen, 2010).

### 4.2 Contexts of transitions

Among the disciplines, the prominent context of transition varied. “Between different contexts” was the most common category in nursing (39.6%), social work (42.9%) and in interdisciplinary research (31.6%), whereas “in daily life” dominated occupational therapy (35.9%). In oral health, almost two out of three studies concerned transition in “outpatient care” (Table 1). To further understand how the concept of transition is used, these three most commonly used contexts are elaborated for the studied disciplines.

#### 4.2.1 Between different contexts

Social work included most articles focusing on transition between contexts. Most of these articles concerned youngsters or young adults and leaving care; “leaving care” could mean the transition between foster care and residential care or between out‐of‐home care and independent living (Daining & DePanfilis, 2007; Höjer & Sjöblom, 2010), or it could mean young people's transition to homelessness forced by a premature home‐leaving (Mayock, Corr, & O'Sullivan, 2011). Such situational changes were sometimes explicitly described as double transitions, that is, between two contexts and at the same time a transition to adulthood. In health and illness, social workers often had the role of facilitator for young patients during the transition from paediatric to adult medical care (Shanske, Arnold, Carvalho, & Rein, 2012) and for older people's transition from hospital to home (Watkins, Hall, & Kring, 2012).

For nursing, transition between contexts mainly was related to transfers in the hospital. Examples are room neonatal intensive care to a special care nursery, that is the transition from curative to palliative care (Thompson, McClement, & Daeninck, 2006) and the transition from paediatric care into adult care in special units, such as diabetes care (Scott, Vallis, Charette, Murray, & Latta, 2005) and kidney transplantation (Remorino & Taylor, 2006). Other transitions described in nursing were moving from institutional care to community care (Fields, Anderson, & Dabelko‐Schoeny, 2011). For instance, communication and coordination were studied during the transition of older persons between nursing homes and hospitals (Kirsebom et al., 2013). Becoming a nurse (Costa, Merighi, & Jesus, 2008) or advancing in the nursing career (Delaney & Piscopo, 2007) was other transitions between contexts.

In occupational therapy, transition between contexts concerned, for instance, the retirement process, that is from working life to retirement (Jonsson, 2011). Other examples were the transition from secondary education into further education and/or payed employment for young adults with physical disabilities (Bjornson, Kobayashi, Zhou, & Walker, 2011) and the transition from hospital to home (Brauer et al., 2011).

For oral health, only one article was found highlighting the transition from working life to retirement and its effects on dental care use (Manski et al., 2011).

#### 4.2.2 In daily life

Daily life included transitions at home and at the workplace. For occupational therapy, the context in daily life was the most common. For instance, Suto (2009) studied migrant women and resettlement, and Sabata, Bruce, and Sanford (2006) investigated home health discharge planning for transition to work for people with disabilities.

In nursing, the most common transition in daily life considered working life; this transition could include the move from the status of a recent graduate to that of an experienced nurse (Lee & Carter, 2012; Tapping, Muir, & Marks‐Maran, 2013) and it could include a promotion or a changed role during a transfer from one professional role to another (Robinson, Kellett, King, & Keating, 2012; Schmitt, 2006).

In interdisciplinary research, transition in daily life mostly concerned the management of long‐term diseases such as diabetes (Rasmussen, O'Connell, Dunning, & Cox, 2007), the transition to adulthood with disabilities or diseases (Magill‐Evans, Wiart, Darrah, & Kratochvil, 2005), or the transition from pregnancy to postpartum (Olshansky & Sereika, 2005).

In social work, transitions in daily life dealt with developmental or relational changes connected to social work practice or in social problematic situations, such as challenges for adoptive parents due to unplanned contacts with the birth family (MacDonald & McSherry, 2013) and unsupported transitions to independent living for young people due to parental substance abuse (Wilson et al., 2008).

For oral health, two different contexts in daily life were found, one concerning the volume of dental care in relation to self‐reported oral health changes (Crocombe, Brennan, & Slade, 2013) and the other highlighting transitions after oral health recommendations based on a theoretical oral health behaviour change model (Schüz, Sniehotta, Mallach, Wiedemann, & Schwarzer, 2009).

#### 4.2.3 Outpatient care

Transition in outpatient care for oral health most often considered changes after different oral health treatments, described by self‐reported oral health and oral health‐related quality of life (Allen, O'Sullivan, & Locker, 2009; McMillan et al., 2005) and the use of dental service and settings (Anderson, Thomas, & Phillips, 2005; Brennan et al., 2012).

Regarding nursing, transitions in outpatient care concerned a change in care, such as transferring youths with diabetes mellitus to adult care (Nakhla, Daneman, To, Paradis, & Guttmann, 2009), transitions of care as experienced by close relatives of traumatic brain injury (Engström & Söderberg, 2011) or chronic illnesses (Wong et al., 2010).

In interdisciplinary research, outpatient care investigated transitions from paediatric care to adult care for cancer survivors (Granek et al., 2012) and youths with cystic fibrosis (Iles & Lowton, 2010), sexual desire during menopausal transition (Woods, Mitchell, & Smith‐DiJulio, 2010) and professional role transitions in primary care (Holt, 2008).

In social work, outpatient care usually concerned social worker's roles in transitions of care, such as the transition to adult care for young people living with HIV (Fair, Albright, Lawrence, & Gatto, 2012) or social work interventions for older people discharged from an acute care setting to home (Fabbre, Buffington, Altfeld, Shier, & Golden, 2011). In one case, outpatient care referred to social services and the transition to fatherhood for young male service users (Reeves, 2006).

For occupational therapy, transition in outpatient care was only found in one article evaluating information of self‐care needs for patients at home after stroke (Cook et al., 2006).

### 4.3 Who underwent the transition?

Regarding who went through the transition, the patient–client was the most common category in all disciplines, except for social work (Table 1). In interdisciplinary studies, this was the case in eight out of ten articles, while about half of the studies for oral health, occupational therapy and nursing belonged to this category.

In interdisciplinary studies, patients‐clients underwent the youths' transition to adulthood (Ruck & Dahan‐Oliel, 2010), elders transition in changing housing (Choi, 2004; Heliker & Scholler‐Jaquish, 2006), menopause transition (Woods et al., 2010) and palliative care patients' experiences (Larkin, De Casterlé, & Schotsmans, 2007).

In oral health, patients reporting outcomes using different dental services (Anderson et al., 2005; Brennan et al., 2012), patients' experiences of oral health related to quality of life after dental treatments (Allen, Savadatti, & Gurmankin Levy, 2009; Tsakos et al., 2010) and changes in dental coverage status in relation to oral status (Manski et al., 2011) were found.

For occupational therapy, the patient‐client experienced, for example, quality in rehabilitation (Lindahl, Hvalsoe, Poulsen, & Langberg, 2013), accommodation transitions for individuals with acquired brain injury (Sloan, Callaway, Winkler, McKinley, & Ziino, 2012), occupational transitions after injury or disease (Shaw, MacAhonic, Lindsay, & Brake, 2009; Švajger & Winding, 2009), mental illness (McKay, 2010) and transition from the hospital to home care (White, Magin, & Pollack, 2009).

In social work, the “person” was the predominating category, followed by “the professional” and “several perspectives.” The “person” as a category most often referred to young adults who were, or had been in, out‐of‐home care, such as foster care (Collins, Spencer, & Ward, 2010), followed by young people in different types of socially difficult situations, such as homelessness (Mayock et al., 2011). Transitions were also viewed both from clients (mostly youth) and professionals (Tilbury, Creed, Buys, & Crawford, 2011).

In nursing, almost three of ten studies concerned the transition of professionals, which was rarely or never the case in the other disciplines. Often, this regarded the transition from nursing student to staff nurse (Pearson, 2009; Walker, Earl, Costa, & Cuddihy, 2013) or from one nursing role to another (Delaney & Piscopo, 2007; Robinson et al., 2012). Moreover, in nursing, 8.5% of those who performed the transition were relatives. This category was rarely mentioned in other disciplines. In occupational therapy, students and employees/job seekers (12.8% for each group) more often were those who performed the transition compared with those in other disciplines (Table 1).

### 4.4 From which perspective was transition seen?

Depending on the disciplines, the perspectives on transition varied. Still, in total, the viewpoint most often was from the patient's/client's perspective (28.0%) followed by the person's perspective (13.1%) (Table 1).

In occupational therapy, the patient/client perspective and the professional perspective were almost equal, while in oral health, more than half of the studies (54.5%) considered the patient/client perspective. This perspective was the most common also in interdisciplinary, although to a less degree (38.2%). In nursing, however, the professional perspective was twice as common as the second most used perspective, which was from the patient's/client's point of view. In social work, transition from the perspective of the person was used in 38.1% of the studies. In oral health, studies focused on the patients' view of oral health (McMillan et al., 2005), oral treatments (Allen, O'Sullivan et al., 2009; Anderson et al., 2005), dental service and coverage (Manski et al., 2011).

In occupational therapy, transition viewed from the patient/client's perspective considered, for instance the clients' perspective on the return to work (Bergmans et al., 2009; Švajger & Winding, 2009), recovery patterns (Prvu Bettger, Coster, Latham, & Keysor, 2008) and the experience of mental illness (McKay, 2010). Transition viewed from the professional perspective in this discipline focused on home‐based rehabilitation (Cook et al., 2013) and school involvements (Michaels & Orentlicher, 2004).

In interdisciplinary studies, articles taking the patient's/client's perspective explored, for instance, the menopause transition (Smith‐DiJulio, Woods, & Mitchell, 2008), patients' transition between care settings (Toscan, Mairs, Hinton, Stolee, & Team, 2012) or transitions to a community‐based setting (Nishita, Wilber, Matsumoto, & Schnelle, 2008).

In social work, many studies taking the perspective of the person concerned young people in out‐of‐home care or the leaving care processes (Reimer, 2010). In many of these cases, the youngsters went through a double transition as their living conditions changed in parallel with their transition from childhood to “instant adulthood” (Rogers, 2011).

In nursing, compared with other disciplines, the perspective was more often taken from the professionals and focused mostly on the transition from nursing student to occupational nurse (Goodwin & Candela, 2013), as well as when shifting from one role to another, such as becoming CEO/president of their organization (Patton, 2012) or becoming a nurse specialist (Robinson et al., 2012).

Of those patients/clients who underwent the transition (*N* = 209), the transition was regarded from the patient's/client's perspective in 45% of the cases (not shown in Table 1). This relationship was the case in the disciplines of nursing (43.1%), occupational therapy (47.6%) and interdisciplinary research (44.2%). In oral health, most articles (85.7%) showed this relationship, whereas for social work, the corresponding figures were 20%.

The transition of patients/clients was regarded from a professional perspective in almost one fourth (22%) of the studies (not shown in Table 1). In social work, all these studies considered the social worker's role in clients' transition in an institutional framework and focused on interventions (Fabbre et al., 2011), procedures (Höjer & Sjöblom, 2011) or assessments (B. R. Lee, Shaw, Gove, & Hwang, 2010).

Among all examined articles (*N* = 350), 37 reported on the professional who underwent the transition (not shown in Table 1). In 95% of these, the transition was also described from a professional perspective. All except three were found in nursing.

## 5 DISCUSSION

This analysis continues the quest of Kralik et al. (2006) beyond the mere scoop of nursing research by exploring how the concept of transition is used in occupational therapy, oral health and social work, as well as in nursing and interdisciplinary studies. The main result is that all included articles, except five, could be categorized according to Meleis et al. (2000) and that all types of transitions were found in all disciplines included, except for development, which was not found for oral health. Those five articles categorized as another typology than those described by Meleis et al. (2000) were not further analysed in our study. However, a new transition typology has recently been found in a review, named as the lifestyle transition (Munck et al., 2018), indicating that the concept of transition in adulthood is developing.

In relation to the literature review by Kralik et al. (2006), our study indicates that the concept of transition has been used in a larger extent during 2003–2013 compared with the previous 10 years. This could be explained by differences in study design. Our study shows that the concept of transition is used in both qualitative and quantitative studies, as well as in theoretical summaries.

In this review, nursing research is the discipline presenting most articles on transition, except for interdisciplinary studies, which, however, also include the research area of nursing. This dominance could be explained by the fact that transition has been used in nursing research for a long time and has developed as an important concept in nursing (Chick & Meleis, 1986; Meleis et al., 2000).

In nursing, the health‐illness type of transition dominates in combination with the transition of patients regarded from the professionals' perspective. This finding is interesting because the notion of healthy transition processes versus unhealthy has been described as the core of the transition and of special concern for nurses (Schumacher, Jones, & Meleis, 1999). Meleis et al. (2000) argued that there are two reasons for this: 1) nurses often are the primary caregivers of people undergoing transitions associated with health problems and 2) nurses tend to be involved in preparing clients for impending transitions and to be those “who facilitate the process of learning new skills related to clients' health and illness experiences” (Meleis et al., 2000, p. 13). Another reflection is that the most recently added typology—organizational transition—constitutes the third largest transition type (13.2%) in nursing, twice as big as the developmental transition. This could be interpreted as a sign of nurses showing greater interest in doing research on organizations and organizational changes, thereby showing the importance to study this level of transition at the individual, couple and family level.

Although Meleis and her colleges introduced and developed the concept of the transition in relation to nursing research and practice, they have presented transition as “a multiple concept embracing the elements of process, time span and perception” (Chick & Meleis, 1986, p. 239). Such broad aspects are useful in various disciplines and with these disciplines, the current study demonstrated how the concept of transition is used in occupational therapy, oral health and social work, as well as in interdisciplinary research in health and welfare. However, the concept is used less in occupational therapy, oral health and social work compared with nursing. This might be a consequence of these disciplines' lack of a developed theory using transition as a core concept. Moreover, in disciplines other than nursing, such as social work, the influence from sociology might imply that other concepts such as career (Davis, 1963; Roth, 1963), change (Watzlawick et al., 1974) and trajectory (e.g., Glaser & Strauss, 1968) are used in parallel to transition.

Although the concept of transition is used in all studied disciplines, there are interesting differences, such as that in oral health, organizational transitions are the second most frequently used type of transition. However, in this discipline, the total number of studies is few and the concept seems to be used more often in the last five years of the study period.

Further, the results show considerable differences between disciplines regarding which contexts the examined transition occurred in, even when the same category was used. For instance, in social work and nursing, the category “between different contexts” was used differently. In nursing, the in‐between stages often appeared in the hospital context, while in social work, “between contexts” reflected the transferal from one “home” to another, commonly referring to young people in foster care or residential treatment. These differences seemed to reflect the different type of work where social workers and nurses are engaged in (for instance child welfare and nursing, respectively). Similarly, the context of “daily life” was most commonly used in occupational therapy and “outpatient care” was most frequent in oral health, corresponding to the various working conditions of these professionals.

The utility of transition, as a concept in research in health and welfare, is further strengthened by how the diversity in professional assignment is also noticeable by the category “who underwent the transition.” For all disciplines in health care (i.e. nursing, occupational therapy and oral health), the “patient” was the one who went through the transition most often. This finding corresponds to these professionals' work in healthcare institutions, such as hospitals or outpatient clinics. In social work, on the other hand, the category of “person” reflects the work with people in the community. Consequently, this study demonstrates the usefulness of the concept of transition for studies in various disciplines which is in line with Kralik et al. (2006, p. 324), who raised the importance of “understanding the transition of the process to assist people to move through it” in a healthy direction. Thus, the use of the concept of transition is important for all professionals to understand various changes in health and well‐being, as well as in relation to professional, personal or organizational development.

Nevertheless, this study also shows an interesting disciplinary difference, which could not be explained from variations of working conditions. Regarding which perspective the transition was viewed, the “patient/client” or the “person” was the most prominent in occupation therapy, oral health and social work. However, in nursing research, the transition process was regarded mainly from the point of view of the professional. This self‐reflecting focus on the nurse as a professional could not be understood clearly by the specific working conditions for nurses compared with other professionals. However, it might be explained by the long tradition of using transition in research and, in particular, the concept's usefulness for providing “directions for nursing practice,” as well as for the “development of nursing therapeutics” (Im, 2010, p. 425). In other studied disciplines, such a theoretical framework is lacking.

### 5.1 Limitation

Adoption of a systematic review approach enhances the scientific rigour of a review. However, this review was performed by systematic steps guided by a theoretical framework and not a systematic review, which may be a limitation. Several researchers from different disciplines were included in the analysis, which could be seen as a possible limitation concerning inter‐reliability, but also a strength related to the study aim. Another possible limitation is that only four disciplines are included in the study. In health and social welfare, also other disciplines such as medicine, psychology and social pedagogy could have been considered. This review included articles published at latest 2013, an extension by including also the more recent years would probably have further enhanced the value of the analysis. At the same time, our study points to the need to continuously study concepts such as transition to investigate how it develops in disciplines where the term is well established as well as its dissemination to and use in other subject fields such as medicine and psychology to name a few important disciplines in health and social welfare not yet included in reviews on the concept of transition.

## 6 CONCLUSIONS

The results from this study show that although there were differences between various disciplines, few articles could not be categorized in the four types of transitions identified and explored in nursing. Further, the most recently added typology—organizational transition—was in our data and was used almost as much as developmental transition. This outcome indicates that the decision to extend the concept with the “new” typology was well founded.

Our main conclusion is that the concept of transition appears useful in health and social welfare research in a broad perspective and in diverse ways. This implies its flexibility but also the usefulness of the concept to explore various changes.

## CONFLICT OF INTEREST

No conflict of interest has been declared by the authors.

## APPENDIX 1

A.D.U.L.T. (Adulthood, Daily‐Life, Utility, Life‐style, Transition) research group, here including the following members: Inger Ahlstrand PhD, Professor Anders Broström, Associate Professor Eleonor I. Fransson, Professor emeritus Bengt Fridlund, Nina Gunnarsson PhD, Associate Professor Maria Henricsson, Associate Professor Sofia Kjellström, Associate Professor Anna Sandgren, Center for Collaborative Palliative care, Department of Health and Caring Sciences, Linnaeus University, Växjö, Sweden.
